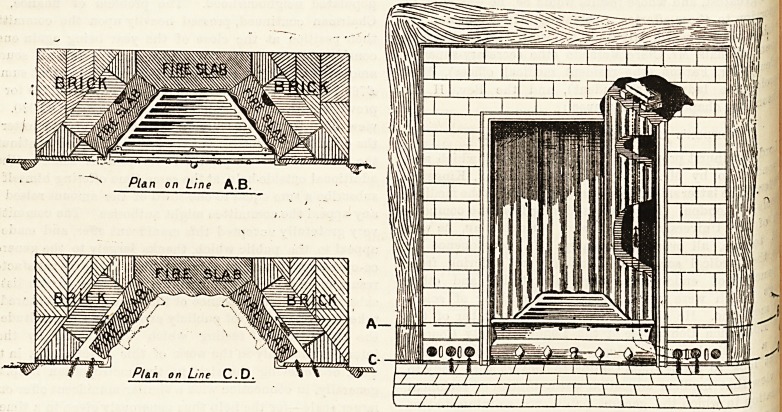# Practical Departments

**Published:** 1904-05-07

**Authors:** 


### PRACTICAL DEPARTMENTS.

### "TROPICAN" GRATES.

We have repeatedly pointed out in our columns that there is a considerable difference in the behaviour of ordinary domestic grates, and in this fact we are confirmed by the Coal Smoke Abatement Society, who have recently made various careful tests. We would point out that it is quite possible to combine heating power and economy of fuel with a comparative freedom of smoke after a fire has been properly lighted, and a very good example of a grate which fulfils these conditions is the "Tropican," the subject of our illustration this week. In the case of this patent grate, manufactured by Messrs. Chavasse and Kerr, of Birmingham, we find some interesting points, and perhaps the two most important are as follows:—(No. 1) Special air-ducts for the purpose of heating and rarefying the

Plan on Line A.B.

Plan on Line C.D.

A—
C—

air taken from the floor-level of the room, and discharging it into the chimney breast in a steady column above the fireplace, while reserving the main opening for radiating purposes, and (2) an opening of large dimensions which increases the value of the heating surface, having special regard to the former condition (No. 1). In practice the "Tropican" should work with success, but we would like to see some means adopted for cleaning the diminutive air-ducts in an efficient manner, for upon the clearness of these the success of the whole system depends. We would suggest in this case small trays extending to the bases of the little shafts behind the fireclay backing. The ends of these trays could contain the holes required for ventilation, and they could be readily pulled out after a wire brush had been pushed through, and down from the upper apertures. The agents for these fireplaces in London are The Hardware Trading Co., of No. 5 New Compton Street, Charing Cross Road.

## Figures and Tables

**Figure f1:**